# Willingness to decrease mammogram frequency among women at low risk for hereditary breast cancer

**DOI:** 10.1038/s41598-019-45967-6

**Published:** 2019-07-03

**Authors:** Yue Guan, Eric Nehl, Ioana Pencea, Celeste M. Condit, Cam Escoffery, Cecelia A. Bellcross, Colleen M. McBride

**Affiliations:** 1 Department of Behavioral Sciences and Health Education, Rollins School of Public Health, Emory University, Atlanta, USA; 2 Department of Communication Studies, Franklin College of Arts and Sciences, University of Georgia, Athens, USA; 3 Department of Human Genetics, Emory University School of Medicine, Atlanta, USA

**Keywords:** Population screening, Risk factors

## Abstract

This study aimed to assess women’s willingness to alter mammogram frequency based on their low risk for HBOC, and to examine if cognitive and emotional factors are associated with women’s inclination to decrease mammogram frequency. We conducted an online survey with women (N = 124) who were unlikely to have a *BRCA* mutation and at average population risk for breast cancer based on family history. Most women were either white (50%) or African American (38%) and were 50 years or older (74%). One-third of women (32%) were willing to decrease mammogram frequency (as consistent with the USPSTF guideline), 42% reported being unwilling and 26% were unsure. Multivariate logistic regression showed that feeling worried about breast cancer (Adjust OR = 0.33, p = 0.01), greater genetic risk knowledge (Adjust OR = 0.74, p = 0.047), and more frequent past mammogram screening (Adjust OR = 0.13, p = 0.001) were associated with being less willing to decrease screening frequency. Findings suggest that emerging genomics-informed medical guidelines may not be accepted by many patients when the recommendations go against what is considered standard practice. Further study of the interplay between emotion- and cognition-based processing of the HBOC screen result will be important for strategizing communication interventions aimed at realizing the potential of precision public health.

## Introduction

Evidence-based guidelines now endorse using genetics-informed assessments to stratify populations on cancer risk^1^. The rationale is that such risk information enables a veritable “win-win” that enables prevention and early detection strategies to be tailored to improve health outcomes while reducing patient burden and health care costs. Concurrently, the US Preventative Services Task Force (USPSTF) has updated screening mammography recommendations holding that asymptomatic individuals who are at low risk for familial breast cancer syndromes (such as hereditary breast and ovarian cancer syndrome [HBOC]) begin mammogram screening at age 50 and that women have biannual mammograms^2,3^. Similarly, the American Cancer Society (ACS) announced in 2015 that, it had modified its recommendations to align more closely with the USPSTF^4^. ACS’s stated rationale was that the modification better balanced the potential benefit of early detection against the now-clearer evidence about the risks of false alarms and overtreatment^5^.

Brief family history screening tools have been scientifically validated for use to stratify risk^1^ and provide guidance regarding who should get a mammogram and when. Implementation of these risk assessment tools at the population level as supported by the CDC Tier 1 evidence^6^ will identify the majority of women to be at low genetic risk. To date, no one has considered whether HBOC screening could be a “teachable moment” for encouraging the adoption of risk-stratified screening guidelines, particularly for women at low hereditary risk. Understanding how the majority of women who participate in HBOC screening perceive the implications of their results as they relate to breast cancer screening could have significant public health impact.

Despite the established utility of mammography screening for early detection of breast cancer, mammograms can yield false positive results that produce unwarranted fear and psychological stress, and expose women to unnecessary treatment, pain, side effects, and health care costs^7^. Half of all women getting annual mammograms beginning at age 40, over a 10-year period will be called back for what is determined to be a false positive finding^8^. Additionally, societal health care costs attributed to annual mammography were estimated at 7.8 billion dollars in 2010^9^. Screening frequency is one of the major drivers of these costs; adoption of USTPSTF guidelines would reduce these costs by half to 3.5 billion^9^. Thus, increasing breast cancer screening intervals for those not deemed to be at high inherited risk could reduce adverse consequences and lower health care costs^10–14^.

Convincing women at low risk for HBOC that they could start screening at a later age or decrease the frequency of their mammograms will likely be very challenging, despite the sizeable risk-benefit tradeoff related to the frequency of screening. As evidence, mammography rates did not decrease following the release of the 2009 USPSTF guideline supporting less frequent mammograms starting at age 50^15–17^. Women did not view these guideline changes favorably, and reacted to reductions in mammography frequency with feelings of distrust as they felt the guideline change was driven by cost saving efforts on the part of insurance companies as opposed to improving their health^18,19^. This may be particularly true for women from minority groups who have experienced health care discrimination, commonly report lower trust in health care systems^20^ and are more skeptical of genomics^21^. Although many women had stories of anxiety and discomfort related to mammograms, the majority of women stated that they intended to continue with annual screenings, with some wanting screenings more than once a year from an earlier age^18^. While screening practices that are contrary to guideline recommendations are common, the determinants of patient receptivity to shifting mammography screening frequency have yet to be explored.

Conceptual models of information processing can provide a framework for considering factors that might influence willingness to reduce mammography frequency. These models suggest both cognitive and emotional factors that together influence how individuals respond to risk information^22,23^. Important cognitive factors include risk-related knowledge, perceived salience and reliability of information. This level of processing is influenced in turn, by emotional factors such as worry and fear that can motivate self-protective reasoning and influence how risk information is interpreted^22^. Thus, consideration of these factors will be critical to any efforts to engage women to thoughtfully consider decreasing mammography screening.

To this end, we conducted an exploratory study aimed to assess women’s willingness to alter mammogram frequency based on their low likelihood to be at risk for HBOC, and to examine if individual cognitive and emotional factors suggested by information processing frameworks are associated with women’s inclination to decrease mammogram frequency.

## Materials and Methods

### Pilot study design and population

This pilot study was a substudy of a larger project aimed at increasing identification and referral of women at highest risk for HBOC to genetic services. Women were approached in the waiting rooms of three Emory clinic breast imaging centers to complete an HBOC screening tool between April 2016 and June 2017. The Breast Cancer Genetics Referral Screening Tool version 3.0 (B-RST^TM^ 3.0, www.brcagenescreen.org) was used^24^. B-RST^TM^ has been endorsed by the USPSTF as one of several validated screening tools that are clinically useful for estimating the probability of *BRCA1/2* mutations and identifying women who should be referred for cancer genetic counseling^1^. This tool uses a validated algorithm which includes breast cancer (<40 years old, 40–50 years old, >50, years old), bilateral breast cancer, ovarian cancer, male breast cancer and Ashkenazi Jewish ancestry in 1^st^ and 2^nd^ degree relatives^24–26^. It should be noted that the B-RST^TM^ does not incorporate other breast cancer risk factors (e.g., smoking, alcohol consumption, age at menarche, age at first live birth of child) for breast cancer risk assessment. The B-RST^TM^ provides two categories of risk results for *BRCA1/2* mutation based on family history: “positive” and “negative”. Individuals who screen “positive” are at heightened risk for carrying a *BRCA1/2* mutation and directed to receive genetic counseling. The “negative” category is further sub-divided into “negative-average risk” and “negative-moderate risk”, reflective of the expected familial risk for breast cancer aside from carrying a mutation in *BRCA1/2*. Women who screened as negative-average risk were told that they were unlikely to carry a *BRCA1/2* mutation and were at average population risk for breast cancer based on family history. Those who screened as negative-moderate risk were told they were at low risk (less than 5%) of carrying a *BRCA1/2* mutation, and at greater than average population risk for breast cancer.

Our substudy focused on women who received a negative-average B-RST^TM^ screening result. Women with negative-average results were chosen as they represent the group who could benefit from increasing age at initiation and reducing mammography frequency. Women were recruited using a recruitment flyer included in the resource handout materials that were given to patients immediately after completing the B-RST^TM^. The flyer explained briefly that women would be asked to complete a one-time online 20-minute survey regarding understanding of the screen result. Those who were interested were asked to provide an email address to the research team. In addition, all eligible women who consented to be recontacted received an email approximately 2–6 weeks after receiving their B-RST^TM^ results, with an invitation to consent and participate in the online survey. Participants received a $25 Amazon electronic gift card via email upon completion of the survey.

A total of 3,883 women were approached to complete the B-RST^TM^ and 2,429 (62.6%) completed the screening. Among 1,768 women who screened negative (1461 negative-average risk and 307 negative-moderate risk), about 500 were contacted for the online survey. 124 women with a negative-average risk result completed the online survey. All research activities were reviewed and approved by Institutional Review Boards at the Emory University.

### Measures

Willingness to decrease mammogram frequency was assessed with the item “How willing would you be to have mammography screening less often if you were found to be at lower genetic risk of breast cancer based on the B-RST^TM^ result?” Possible responses were: very unwilling, unwilling, not sure, willing, very willing. Participants’ responses were categorized into willing, unwilling, and unsure.

#### Cognitive factors

Perceived need for biannual mammogram: Participants were provided with the stem question, “Based on what you know about mammography screening recommendations for your age” and asked to endorse the recommended mammography frequency: biannual, annual, depends on doctor recommendation, and I don’t know. Women’s perceived need for biannual mammogram was calculated as the agreement between their understanding of mammogram frequency and the USPSTF guideline (biannual for low risk women). Provider perceived need for biannual mammogram was assessed from the patient perspective using one 5-point Likert item, “My health care provider thinks that I should have a mammogram every two years.”

Risk perception: Relative risk perception was assessed by the item “compared to the average woman your age, would you say that you are less likely/as likely/more likely to develop breast cancer.” Risk perception alignment was the 100% agreement of women’s perceived risk with their B-RST^TM^ risk estimation: “negative-average risk” (as likely as the average woman).

Knowledge: Breast cancer genetic knowledge was assessed using a validated scale developed by Lerman^27^ and Erblich^28^. It includes seven items with response options of “true”, “false” and “don’t know” to questions such as: “Ovarian cancer and breast cancer in the same family can be a sign of an inherited *BRCA1* or *BRCA2* mutation.” Recall of B-RST^TM^ result was scored as correct or incorrect and calculated as the concordance of participants’ self-reported screen result and their actual result. Understanding of results (correct/incorrect) was assessed by two items regarding the likelihood of carrying a *BRCA1/2* mutation and the likelihood of breast cancer based on family history. B-RST^TM^ result acceptance was assessed by a 9-item 5-point Likert scale^29^, where participants were asked to respond to statements including “The information I received from the B-RST™ result about my risk for hereditary breast and ovarian cancer seems accurate” and “The information I received from the B-RST™ result was missing some important information about me or my family” (reverse-scored). Sum scores range from 0–45, with a higher score indicating a higher level of acceptance (Cronbach’s Alpha = 0.79).

Trust: Trust in health care provider was measured on a 5-point Likert scale using five items from the multidimensional trust in health care systems scale^30^. Participants were asked to respond to statements including “My health care provider will do whatever it takes to give me the medical care that I need” (Cronbach’s Alpha = 0.96). Trust in breast cancer screening guidelines was measured on a 5-point Likert 5-item scale. Participants were asked to “think about national recommendations for breast cancer screening” and indicate whether they agreed or disagreed with statements such as: “The recommendations for breast cancer screening I have heard are trustworthy” (Cronbach’s Alpha = 0.92).

#### Emotional factors

Cancer worry: Women were asked how often they worried about getting breast cancer with response options from “rarely or never” to “all the time” on a 4-point Likert scale^31–33^.

Negative affect: A shortened 5-item Positive and Negative Affect Schedule (PANAS) scale^34^ was used to measure emotions that might be generated by breast cancer risk information. Women were provided with the stem question, “When I think about breast cancer” and then asked to endorse the extent to which they felt: anxious, calm (reverse coded), upset, confident (reverse coded), and uneasy (Cronbach’s Alpha = 0.89). Total score ranges from 0 to 5; a higher score indicates more negative affect of breast cancer.

#### Mammogram history

Reason for current mammogram: Participants were asked to indicate the reason for their mammogram at the time of the B-RST^TM^ screen. Possible responses were: personal choice for routine screening and doctor recommendation for routine screening.

Past mammogram frequency: Women were asked to report how frequently they had a mammogram in the past two years with answers grouped to “biannually or less” (consistent with USPSTF guideline) or “annually or more.”

#### Demographics

Participants age, race, level of education, and annual household income were measured using items developed from the U.S. Census guidelines^35^. We used a dichotomized age variable (<50 years, >=50 years) to reflect the USPSTF mammogram screening guideline^2^. Health literacy was assessed by a single item, “How confident are you filling out medical forms by yourself?” This has been shown to be accurate in detecting inadequate health literacy skills^36,37^.

### Statistical analysis

Survey responses were collated into Microsoft Excel from REDCap, and all data analyses were performed using STATA Version 15.0 (STATA Corp, College Station, Texas, USA). Descriptive statistics analyzed the demographic and outcome variables to describe the study participants. There was no significant difference between the “unwilling” and “unsure” group in the following patient factors: patient socio-demographic characteristics (age, race, education, income, health literacy), emotional factors (cancer worry and negative affect), cognitive factors (risk perception, genetic risk knowledge, B-RST^TM^ result recall and understanding, trust in health care), and mammogram history (reason for current mammogram, past mammogram frequency, and patient and provider perceived need for biannual mammogram). To increase power, we combined the “unwilling” and “unsure” groups to be one group, “not willing,” in subsequent logistic regression analyses with “not willing” as the reference category. The dichotomized outcome, willing vs. not willing to decrease screening frequency, was regressed on each of the patient factors separately. In all analyses, 2-tailed tests and p-values < 0.05 were used to draw conclusions regarding statistical significance.

### Ethical approval

All procedures performed in studies involving human participants were in accordance with the ethical standards of the institutional and/or national research committee and with the 1964 Helsinki declaration and its later amendments or comparable ethical standards.

### Informed consent

Informed consent was obtained from all individual participants included in the study.

## Results

### Participants demographics and mammogram history

The majority of negative-average risk women (i.e., unlikely to have a BRCA mutation and at average population risk for breast cancer based on family history) were either white (50%) or African American (38%) and were 50 years or older (74%) (Table 1). About one-third of women reported being extremely confident to fill out medical forms, an indicator of high health literacy. The majority of women reported at least college education (75%) and half (50%) reported annual household incomes greater than $75 K.

**Table 1:** Descriptive results of patient characteristics by women’s willingness to decrease mammogram frequency.

Patient factors	All (N = 124)	Willingness to decrease mammogram frequency		
		Yes (n = 40)	No (n = 52)	Unsure (n = 32)
**Socioeconomic characteristics**				
Age, mean (SD, range)	57.7 (10.5, 34–87)	58.7 (11.6)	55.9 (10.4)	59.2 (9.1)
<50 years	32 (26%)	9 (23%)	17 (33%)	6 (19%)
>=50 years	92 (74%)	31 (77%)	35 (67%)	26 (81%)
Race				
White	62 (50%)	20 (50%)	24 (46%)	18 (56%)
African American	48 (38%)	13 (33%)	25 (48%)	10 (31%)
Other (e.g., Asian, American Indian, Not specified)	14 (12%)	7 (17%)	3 (6%)	4 (13%)
Education				
Some college or less	26 (25%)	6 (19%)	14 (33%)	6 (21%)
College graduate	34 (33%)	7 (23%)	18 (42%)	9 (31%)
Graduate or professional degree	43 (42%)	18 (58%)	11 (25%)	14 (48%)
Household Income				
Less than $50,000	27 (29%)	7 (26%)	12 (32%)	8 (29%)
$50,001-$75,000	20 (21%)	5 (19%)	9 (24%)	6 (21%)
$75,001 and over	46 (50%)	15 (55%)	17 (44%)	14 (50%)
Health Literacy				
Less than extremely confident	15 (12%)	7 (17%)	4 (8%)	4 (13%)
Extremely confident	44 (35%)	13 (33%)	20 (38%)	11 (34%)
Do not wish to answer	65 (53%)	20 (50%)	28 (54%)	17 (53%)
**Emotional factors**				
Breast cancer worry				
No worry	58 (47%)	26 (65%)	18 (35%)	14 (44%)
Any worry	66 (54%)	14 (35%)	34 (65%)	18 (56%)
Breast cancer negative affect, mean (SD, range)	14.5 (4.8, 5–24)	2.9 (1.1)	3.1 (1.3)	3.1 (1.2)
**Cognitive factors**				
Relative risk perception				
More likely	7 (6%)	1 (2%)	6 (8%)	0
Less likely	53 (45%)	17 (45%)	22 (46%)	14 (47%)
As likely (Consistent with B-RST^TM^ estimate)	58 (49%)	20 (53%)	22 (46%)	16 (53%)
Risk perception alignment with B-RST^TM^ estimate				
Inconsistent (more or less likely)	66 (53%)	18 (47%)	28 (54%)	14 (47%)
Consistent (as likely)	58 (47%)	20 (53%)	22 (46%)	16 (53%)
Genetic risk knowledge, mean (SD, range)	4.0 (1.4, 0–7)	3.6 (1.3)	4.2 (1.3)	4.3 (1.7)
B-RST^TM^ result recall				
Wrong	63 (54%)	20 (56%)	26 (52%)	17 (57%)
Correct	53 (46%)	16 (44%)	24 (48%)	13 (43%)
B-RST^TM^ understanding of BRCA mutation risk				
Wrong	53 (45%)	19 (51%)	20 (40%)	14 (47%)
Correct	64 (55%)	18 (49%)	30 (60%)	16 (53%)
B-RST^TM^ understanding of general population risk				
Wrong	72 (62%)	28 (76%)	27 (55%)	17 (57%)
Correct	44 (38%)	9 (24%)	22 (45%)	13 (43%)
B-RST^TM^ acceptance	32.6 (4.9, 22–45)	32.6 (4.8)	32.7 (5.6)	32.2 (3.7)
Trust in health care providers, mean (SD, range)	20.3 (4.2, 5–25)	20.0 (3.9)	20.5 (4.5)	20.5 (4.3)
Trust in screening guidelines, mean (SD, range)	18.9 (3.5, 5–25)	18.9 (2.2)	18.6 (4.5)	19.5 (3.1)
**Mammogram history**				
Reason for current mammogram				
Personal choice for routine screening	42 (36%)	10 (29%)	19 (38%)	13 (41%)
Doctor recommendation for routine screening	75 (64%)	25 (71%)	31 (62%)	19 (59%)
Past mammogram frequency				
Biannually or less (consistent with USPSTF guideline)	18 (14%)	13 (33%)	2 (4%)	3 (9%)
Annually or more	105 (86%)	27 (67%)	49 (96%)	29 (91%)
Understanding of mammogram screening frequency based on age				
Biannual (consistent with USPSTF guideline)	13 (10%)	3 (7%)	5 (10%)	5 (16%)
Annual	82 (67%)	25 (63%)	35 (67%)	22 (71%)
Depend on doctor recommendation	20 (16%)	8 (20%)	8 (15%)	4 (13%)
I don’t know	8 (7%)	4 (10%)	4 (8%)	0
Women perceived need for biannual mammogram				
Biannual (consistent with USPSTF guideline)	13 (11%)	3 (7%)	5 (9%)	5 (16%)
Other (annual, depend on doctor recommendation, I don’t know)	110 (89%)	39 (93%)	48 (91%)	26 (84%)
Provider perceived need for biannual mammogram				
Do not agree	106 (85%)	32 (80%)	46 (88%)	28 (87%)
Agree	18 (15%)	8 (20%)	6 (12%)	4 (13%)

The reason for women’s current mammogram was for routine screening, either due to personal choice (n = 42) or doctor recommendation (n = 75). Few women reported having had a mammogram biannually or less frequently in the past two years (14%, n = 18), believed that they should have biannual mammogram based on age (11%, n = 13), or agreed their doctor would support biannual mammogram (15%, n = 18).

### Willingness to change the frequency of mammograms

One third (n = 40) of women reported being willing to decrease screening (as consistent with the USPSTF guideline), 42% (n = 52) reporting being “unwilling” and 26% (n = 32) were “unsure” about decreasing mammogram frequency.

### Emotional factors related to breast cancer

Over half (54%) of women reported feeling worried at least sometimes about getting breast cancer. Breast cancer-related negative affect was moderate (mean = 14.5, range: 5–24). In particular, more than one-third women agreed or strongly agreed that they felt uneasy (48%), or anxious (44%) when they thought about breast cancer.

### Cognitive factors related to breast cancer

Half of the women perceived themselves to be as likely to develop breast cancer as other women their age (as depicted on their actual B-RST^TM^ result) while 44% indicated they were at lower risk and 6% indicated they were at higher risk.

Women demonstrated a moderate level of knowledge related to factors that influence breast cancer risk (mean = 4.0, SD = 1.5, range: 0–7). The majority of women responded correctly that: *“ovarian cancer and breast cancer in the same family can be a sign of an inherited BRCA1/2 mutation”* (78%), *“there are many different genes that when altered can increase cancer risk”* (80%), and *“a woman who does not have a BRCA1/2 gene mutation can still get breast or ovarian cancer”* (79%). However, most women (87%) incorrectly agreed “*that about half of all breast cancers are caused by mutations in BRCA1 and BRCA2”*.

Less than half of women (46%, 53/116) accurately reported their B-RST^TM^ result as “negative-average risk”. Over half of the women (55%, 64/117) correctly interpreted that their negative-screen result indicated it was *unlikely* that they carried a *BRCA1/2* mutation, but fewer women (38%, 44/116) correctly understood that they remained at average risk for breast cancer. B-RST^TM^ result acceptance (based on a 9-item scale – e.g. *“The information I received from the B-RST*^*TM*^ *result about my risk for HBOC seems accurate*”) was high (mean = 32.6, SD = 4.9, range: 22–45).

Women reported relatively high trust in health care providers (mean = 20.3, SD = 4.2, range: 5–25), but their trust in national professional mammogram screening guidelines was relatively lower (mean = 18.9, SD = 3.5, range: 5–25).

### Association between information processing factors and women’s willingness to decrease mammogram frequency

As shown in Table 2, bivariate logistic regressions showed that women who had any worry about breast cancer were less likely to be willing to decrease mammogram frequency, compared to women who reported no worry (OR = 0.33, 95% CI: 0.15–0.73; p = 0.006). Knowledge was negatively associated with willingness to decrease screening frequency; greater knowledge of breast cancer genetic risk (OR = 0.74, 95% CI: 0.57–0.97; p = 0.03) and correct interpretation of the implications of HBOC screen results for breast cancer risk (OR = 0.40, 95% CI: 0.17–0.97; p = 0.04) were associated with not being willing to decrease mammogram frequency. Women who had annual or more frequent mammograms within the past two years (OR = 0.13, 95% CI: 0.04–0.41; p < 0.001) were less likely to be willing to decrease mammogram frequency. Other information processing factors (e.g., trust, acceptance of risk information relevance, risk perception) were not significantly associated with willingness to reduce screening frequency.

**Table 2:** Association between information processing factors and women’s willingness to decrease mammogram frequency: Results from bivariate logistic regressions with “Not willing” as the reference category.

Patient factors	Willing (n = 40) vs Not willing (n = 84)	
	OR (95%CI)	p-value
**Socioeconomic characteristics**		
Age		
<50 years (REF)		
>=50 years	1.30 (0.54–3.14)	0.56
**Race**		
African American (REF)		
White	1.28 (0.56–2.94)	0.56
Other (e.g., Asian, American Indian, Not specified)	2.69 (0.79–9.17)	0.11
Education		
Some college or less (REF)		
College graduate	0.86 (0.25–2.97)	0.82
Graduate or professional degree	2.40 (0.80–7.18)	0.12
**Household Income**		
Less than $50,000 (REF)		
$50,001-$75,000	0.95 (0.25–3.60)	0.94
$75,001 and over	1.38 (0.48–3.99)	0.55
**Health Literacy**		
Less than extremely confident (REF)		
Extremely confident	0.48 (0.14–1.60)	0.23
Do not wish to answer	0.51 (0.16–1.59)	0.25
**Emotional factors**		
Breast cancer worry		
No worry (REF)		
Any worry	0.33 (0.15–0.73)	0.006
Breast cancer negative affect	0.99 (0.91–1.07)	0.73
**Cognitive factors**		
Relative risk perception		
Inconsistent with B-RST^TM^ estimate (REF)		
Consistent with B-RST^TM^ estimate	1.21 (0.57–2.57)	0.62
Genetic risk knowledge	0.74 (0.57–0.97)	0.03
**B-RST** ^**TM**^ **result recall**		
Wrong (REF)		
Correct	0.93 (0.42–2.05)	0.86
B-RST^TM^ understanding of BRCA mutation risk		
Wrong (REF)		
Correct	0.70 (0.32–1.53)	0.37
B-RST^TM^ understanding of general population risk		
Wrong (REF)		
Correct	0.40 (0.17–0.97)	0.04
B-RST^TM^ acceptance	1.00 (0.92–1.09)	0.96
Trust in health care providers	0.98 (0.90–1.07)	0.63
Trust in screening guidelines	1.00 (0.90–1.11)	0.98
**Mammogram history**		
Reason for current mammogram		
Personal choice for routine screening (REF)		
Doctor recommendation for routine screening	1.60 (0.68–3.77)	0.28
Past mammogram frequency		
Biannually or less (consistent with USPSTF guideline) (REF)		
Annually or more	0.13 (0.04–0.41)	<0.001
Women perceived need for biannual mammogram		
Other (annual, depend on doctor recommendation, I don’t know) (REF)		
Biannual (consistent with USPSTF guideline)	0.59 (0.15–2.28)	0.45
Provider perceived need for biannual mammogram		
Do not agree (REF)		
Agree	1.85 (0.67–5.12)	0.24

Although our power was limited, we conducted selected multivariate logistic regression analyses. Significant patient factors identified from the bivariate logistic regressions were included in the final multivariate logistic regression model: cancer worry, genetic risk knowledge, and past mammogram frequency. Results indicated that feeling worried about breast cancer (Adjust OR = 0.33, 95% CI = 0.14–0.77; p = 0.01), greater genetic risk knowledge scores (Adjust OR = 0.74, 95% CI = 0.56–1.00; p = 0.047), and more frequent past mammogram screening (Adjust OR = 0.13, 95% CI: 0.04–0.42; p = 0.001) were associated with being less willing to decrease screening frequency (pseudo r-square = 0.17).

## Discussion

To our knowledge, this is the first study to explore how women screened at low risk of being *BRCA1/2* mutation carriers might respond to recommendations for decreased frequency of mammogram screening. These women make up the majority of women who participate in HBOC screening. Women who receive a “negative-average” HBOC screen result are unlikely to have a *BRCA* mutation and are at average population risk for breast cancer based on family history^24^. National guidelines support initiating mammography at age 50 with biannual screening thereafter^2^. We found that a large proportion of women (86%) in the study had adopted routine annual mammogram screening regimen, which is in excess of guideline recommendation. After learning that they were at lower genetic risk of breast cancer, over half (67%) reported that they were either unwilling or unsure about reducing mammography screening. These preliminary results suggest that proactive efforts would be needed to sway the large proportion of low risk women to consider reducing mammography screening frequency.

We further explained how individual emotional and cognitive factors influence how willing women may be to adjust their screening based on their genetic risk, guided by information processing models^22,23^. This approach was particularly novel, as past studies of “deimplementation” of medical overuse have focused on provider and contextual factors^38,39^ and not on patients’ processes of “unlearning” ineffective health behaviors. Results from this study support some of the proposed relationships, most notably those between breast cancer worry, genetic risk knowledge, past mammogram frequency, and women’s willingness to decrease mammogram screening.

Cancer worry was significantly associated with women being less willing to adopt less frequent mammography screening. The effect of breast cancer worry on screening behavior has been inconsistent; worry may enhance screening^40^ or lead to screening avoidance^41^. Unfortunately, we did not assess worry related to the likelihood of false positive results. Others have shown with a sample of women ages of 40 and 59 that awareness of over-diagnoses (27%) or overtreatment (40%) due to mammography was low^42^. Prior work has also shown that women experience anxiety about guideline changes and prefer not to change the frequency of their use of mammography^18^. Due to the widely reinforced mammogram recommendation as the way to prevent breast cancer, negative emotions associated with breast cancer likely would need to be addressed in helping women consider less frequent mammograms. Future larger studies should include a broader array of emotion-related measures based on information processing frameworks.

Greater genetic risk knowledge related to breast cancer was associated with *not* being willing to decrease mammogram frequency. We evaluated whether heightened knowledge might be due to cancer worry prompting more information seeking or lessened trust. However, we did not find significant associations between knowledge and worry or trust in this population. Unfortunately, the small sample size precluded us from testing moderating and mediating associations. Given these factors, intervention efforts that solely focus on improving the public’s cancer genetic knowledge may not serve the goal of promoting adoption of evidence-based screening guidelines. Understanding how different types of knowledge impact women’s willingness to de-implement, when appropriate, annual mammogram screening based on their genetic risk, and how relevant knowledge interacts with women’s emotional factors, will be critical to facilitate effective decision making for patients.

We also found that women who had annual or more frequent screening in the past were less likely to forgo future mammograms based on learning more about their breast cancer risk. This finding is consistent with previous research^38,43,44^. The interplay between emotion- and cognition-based processing of the HBOC screen result may be particularly important to the mammogram decision against the backdrop of discrepancies in public messages about mammography^45^, the complexity of risk factors of breast cancer, and the uncertainty of potential harms (e.g., likelihood of getting a false-positive mammogram screen result)^46^. Women faced with this discrepant information will likely default to previous screening behaviors rather than to act on uncertain information. It is evident that most women in the study believed that they should have annual mammogram based on age (90%) and did not believe their doctor would support biennial mammogram (85%). In the context of the widely reinforced one-size-fits-all annual mammogram recommendation being deeply ingrained into patients’ and providers’ perceptions of appropriate medical care, and with public health organizations and advocacy groups pushing screening through advertisements and media campaigns^47^, effectively implementing risk stratified mammogram screening recommendations may be challenging.

We acknowledge that our study findings are limited due to small sample and require replication. While it was difficult to assess the actual participation, survey completion of 124 from 500 contacted, while not unusual, may have resulted in a highly selective sample. Accordingly, the sample was relatively homogeneous with respect to education, income, and other demographics. Women who were recruited at mammography clinics in one medical system and responded to the survey may not be generalizable to women outside of this context. The small sample size also limited power to explore associations among patient factors and women’s willingness to decrease mammogram frequency. In addition, it should be noted that the B-RST^TM^ was developed and validated as a tool to identify individuals at increased risk for *BRCA1/2* mutations and it does not incorporate other breast cancer risk factors (e.g., smoking, alcohol consumption, age at menarche, age at first live birth of child). As such, its predictions for breast cancer risk in the average and moderate range are limited to family history-based information. It should be noted however, that the screen result information sheet did describe non-familial risk factors for breast cancer. Women’s perceptions of the need for annual mammograms may have been influenced by these risk factors.

Despite these limitations, our findings show that, while some women at low genetic risk for breast cancer were willing to decrease mammogram frequency consistent with the USPSTF guideline, more were unwilling or unsure about reducing the frequency of mammography. Our sample likely represents the most adherent mammography screening population given that the majority of women adopted routine annual screening schedule. The resistance of guideline change among these well-educated adherent screeners may be suggestive of broader resistance to de-implementing annual mammograms among an average risk population of screeners. The use of screening mammography in excess of guidelines by low risk women could divert health care resources from those who benefit most from screening. Moreover, resulting false positive screening results in unnecessary diagnostic work-up, and biopsies could also divert limited health services^10–14^. To increase the likelihood of adopting genomics-informed medical recommendations, further study of the interplay between emotion- and cognition-based processing of the HBOC screen result will be important, particularly for strategizing communication interventions aimed at realizing the potential of precision public health.

## Data Availability

The authors will make study materials, data and associated protocols promptly available to readers without undue qualifications in material transfer agreements.

## References

[1] Moyer VA (2014). Risk assessment, genetic counseling, and genetic testing for BRCA-related cancer in women: US Preventive Services Task Force recommendation statement. Annals of internal medicine.

[2] Siu AL, Force USPST (2016). Screening for Breast Cancer: U.S. Preventive Services Task Force Recommendation Statement. Ann Intern Med.

[3] Force, U. S. P. S. T. Screening for breast cancer: U.S. Preventive Services Task Force recommendation statement. *Ann Intern Med* **151**, 716–726, W-236, 10.7326/0003-4819-151-10-200911170-00008 (2009). 10.7326/0003-4819-151-10-200911170-00008 19920272

[4] Oeffinger KC (2015). Breast Cancer Screening for Women at Average Risk: 2015 Guideline Update From the American Cancer Society. JAMA.

[5] http://www.berkeleywellness.com/healthy-community/health-care-policy/article/understanding-new-mammogram-guidelines.

[6] Modell Stephen, Greendale Karen, Citrin Toby, Kardia Sharon (2016). Expert and Advocacy Group Consensus Findings on the Horizon of Public Health Genetic Testing. Healthcare.

[7] Tosteson AN (2014). Consequences of false-positive screening mammograms. JAMA Intern Med.

[8] Kerlikowske K (2013). Outcomes of screening mammography by frequency, breast density, and postmenopausal hormone therapy. JAMA Intern Med.

[9] O’Donoghue C, Eklund M, Ozanne EM, Esserman LJ (2014). Aggregate cost of mammography screening in the United States: comparison of current practice and advocated guidelines. Ann Intern Med.

[10] Pashayan N (2011). Polygenic susceptibility to prostate and breast cancer: implications for personalised screening. Br J Cancer.

[11] Darabi H (2012). Breast cancer risk prediction and individualised screening based on common genetic variation and breast density measurement. Breast Cancer Res.

[12] Vilaprinyo E (2014). Cost-effectiveness and harm-benefit analyses of risk-based screening strategies for breast cancer. PLoS One.

[13] Trentham-Dietz A (2016). Tailoring Breast Cancer Screening Intervals by Breast Density and Risk for Women Aged 50 Years or Older: Collaborative Modeling of Screening Outcomes. Ann Intern Med.

[14] Pashayan N, Morris S, Gilbert FJ, Pharoah PDP (2018). Cost-effectiveness and Benefit-to-Harm Ratio of Risk-Stratified Screening for Breast Cancer: A Life-Table Model. *JAMA*. Oncol.

[15] Pace LE, He Y, Keating NL (2013). Trends in mammography screening rates after publication of the 2009 US Preventive Services Task Force recommendations. Cancer.

[16] Dehkordy SF (2015). Trends in Breast Cancer Screening: Impact of U.S. Preventive Services Task Force Recommendations. Am J Prev Med.

[17] Brown C, Nevola A, Martin BC (2018). Lack of Impact of the 2009 USPSTF Guidelines on Rates of Mammography Screening. J Womens Health (Larchmt).

[18] Allen JD (2013). Women’s responses to changes in U.S. Preventive Task Force’s mammography screening guidelines: results of focus groups with ethnically diverse women. BMC Public Health.

[19] Kiviniemi MT, Hay JL (2012). Awareness of the 2009 US Preventive Services Task Force recommended changes in mammography screening guidelines, accuracy of awareness, sources of knowledge about recommendations, and attitudes about updated screening guidelines in women ages 40–49 and 50+. BMC Public Health.

[20] Adams LB, Richmond J, Corbie-Smith G, Powell W (2017). Medical Mistrust and Colorectal Cancer Screening Among African Americans. J Community Health.

[21] Hensley Alford S (2011). Participation in genetic testing research varies by social group. Public Health Genomics.

[22] Tsalatsanis A, Hozo I, Kumar A, Djulbegovic B (2015). Dual Processing Model for Medical Decision-Making: An Extension to Diagnostic Testing. PLoS One.

[23] Griffin RJ, Dunwoody S, Neuwirth K (1999). Proposed model of the relationship of risk information seeking and processing to the development of preventive behaviors. Environ Res.

[24] Bellcross Cecelia, Hermstad April, Tallo Christine, Stanislaw Christine (2018). Validation of Version 3.0 of the Breast Cancer Genetics Referral Screening Tool (B-RST™). Genetics in Medicine.

[25] Bellcross CA, Lemke AA, Pape LS, Tess AL, Meisner LT (2009). Evaluation of a breast/ovarian cancer genetics referral screening tool in a mammography population. Genet Med.

[26] Bellcross C (2010). Further development and evaluation of a breast/ovarian cancer genetics referral screening tool. Genet Med.

[27] Lerman C (1997). Controlled trial of pretest education approaches to enhance informed decision-making for BRCA1 gene testing. J Natl Cancer Inst.

[28] Erblich J (2005). Development and validation of a Breast Cancer Genetic Counseling Knowledge Questionnaire. Patient Educ Couns.

[29] Taber JM (2015). Genetic test reporting enhances understanding of risk information and acceptance of prevention recommendations compared to family history-based counseling alone. J Behav Med.

[30] Egede LE, Ellis C (2008). Development and testing of the Multidimensional Trust in Health Care Systems Scale. J Gen Intern Med.

[31] Karliner LS (2007). Missed opportunities: family history and behavioral risk factors in breast cancer risk assessment among a multiethnic group of women. J Gen Intern Med.

[32] Lerman C (1991). Psychological and behavioral implications of abnormal mammograms. Ann Intern Med.

[33] Lerman C (1991). Psychological side effects of breast cancer screening. Health Psychol.

[34] Thompson ER (2007). Development and validation of an internationally reliable short-form of the positive and negative affect schedule (PANAS). Journal of cross-cultural psychology.

[35] Grieco, E. M. & Cassidy, R. C. *Overview of race and Hispanic origin, 2000*. Vol. 8 (US Department of Commerce, Economics and Statistics Administration, US …, 2001).

[36] Chew LD (2008). Validation of screening questions for limited health literacy in a large VA outpatient population. J Gen Intern Med.

[37] Wallace LS, Rogers ES, Roskos SE, Holiday DB, Weiss BD (2006). Brief report: screening items to identify patients with limited health literacy skills. J Gen Intern Med.

[38] Sharma R (2018). Factors Influencing Overuse of Breast Cancer Screening: A Systematic Review. J Womens Health (Larchmt).

[39] Helfrich CD (2018). How the dual process model of human cognition can inform efforts to de-implement ineffective and harmful clinical practices: A preliminary model of unlearning and substitution. J Eval Clin Pract.

[40] Hay JL, McCaul KD, Magnan RE (2006). Does worry about breast cancer predict screening behaviors? A meta-analysis of the prospective evidence. Prev Med.

[41] Consedine NS, Magai C, Krivoshekova YS, Ryzewicz L, Neugut AI (2004). Fear, anxiety, worry, and breast cancer screening behavior: a critical review. Cancer Epidemiol Biomarkers Prev.

[42] Yu J, Nagler RH, Fowler EF, Kerlikowske K, Gollust SE (2017). Women’s Awareness and Perceived Importance of the Harms and Benefits of Mammography Screening: Results From a 2016 National Survey. JAMA Intern Med.

[43] Lechner L, de Vries H, Offermans N (1997). Participation in a breast cancer screening program: influence of past behavior and determinants on future screening participation. Prev Med.

[44] Mayne L, Earp J (2003). Initial and repeat mammography screening: different behaviors/different predictors. J Rural Health.

[45] Nagler, R. H., Yzer, M. C. & Rothman, A. J. Effects of Media Exposure to Conflicting Information About Mammography: Results From a Population-based Survey Experiment. *Ann Behav Med*, 10.1093/abm/kay098 (2018). 10.1093/abm/kay098 PMC6735717 30596830

[46] Nagler RH, Franklin Fowler E, Gollust SE (2017). Women’s Awareness of and Responses to Messages About Breast Cancer Overdiagnosis and Overtreatment: Results From a 2016 National Survey. Med Care.

[47] Patton, C. Breast Cancer Cause-Marketing: Reworking the Social Contract (2017).

